# Management of the New-Born

**Published:** 1895-01

**Authors:** W. Z. Holliday

**Affiliations:** Augusta, Ga.


### *ORIGINAL COMMUNICATIONS.*

---

### MANAGEMENT OF THE NEW-BORN.*

By W. Z. HOLLIDAY, M.D.,

Augusta, Ga.

There is no greater crisis in the course of human life than that which marks its beginning. None of the lower animals are born so helpless as man. The proper management of the baby at this time is one of the most sacred duties intrusted to the practitioner of medicine. Its importance is not on account of the slender thread by which life is sustained alone, but because, if improper attention is bestowed, the life, though prolonged, may be marred by disease and sorrows which ought to have been avoided. Blind asylums, with their inmates forever shut out from the light of day and the beauties of life, disfigured faces, even when babies are not blind, many of the cripples we see on our streets, the too frequent use of the truss, and the many other witnesses will attest the truthfulness of my statement. These helpless, tender, absolutely dependent embryonic men and women appeal to us in a most pathetic manner to deliver them from these often avoidable misfortunes.

Modern obstetric teachings require that every precaution as to cleanliness or antisepsis shall be observed in the management of labor, and this will aid us in the first care of the baby. When a child has been born at full term, or at any period after the viable age has been reached, it will almost immediately cry out. "With the first inspiration the thorax expands, and air fills the alveoli of the lungs; at the same time the blood passes from the right side of the heart to the capillaries of the pulmonary organs and is returned arterialized to the left side of the heart. As a consequence of the establishment of the pulmonary circulation, the ductus arte-

---

*Read before the Augusta Academy of Medicine, November 7, 1894.

riosus contracts, the foramen ovale closes, and the left ventricle undergoes eccentric hypertrophy." These changes are made necessary, because the important organs of the body can no longer be cared for through the maternal circulation. It has been wisely advised that the child should at once be turned on its right side, because this position favors the closure of the foramen ovale. At the same time its face should be turned from the mother to prevent the discharges from the vagina being received in the face or eyes. In doing this, care should be taken not to pull too much on the cord, as thereby injury may be done to the abdominal walls at umbilicus, and seed sown which, by and by, will produce an umbilical hernia. After the child has respired several times and you are sure the lungs have been fully inflated, the cord may be ligated and cut and the child made to exist as an independent being. Some time before the second stage of labor is concluded the attending physician should have provided himself with a suitable string for ligating the cord. One made of four or six strands of No. 30 spool cotton, which can always be had, answers the purpose very well. The cord should be carefully ligated, using the surgeon's knot, which should be applied, first, two and a half inches from the child's abdomen, then again one inch nearer the maternal end, and then with a pair of scissors a cut must be made between.

Some years ago Dr. Goodell, I think it was, suggested that the cord be stripped, relieving it of the whartonian jelly by pressing it between the thumb and index-finger, thus reducing its bulk, making the ligature more surely retained and causing less of the odor of decomposition when suppuration is taking place. In very fat cords this is a very good rule, but in the average case I have not found it necessary. Again it has been suggested that the maternal end of the cord be not ligated, and claiming that the hemorrhage from it will not be considerable, and that the bleeding by reducing the volume of the placenta causes it to be more readily expelled. I can see no reason why this should be any advantage, so I am accustomed to ligate both ends. Cleanliness seems to demand it.

When the child is the product of a normal birth of a few hours labor, at term, the above course is all that is necessary before proceeding to bathe and dress it. A great many children, however,

(for reasons which need not be enumerated here) are born asphyxiated and apparently, or really, still-born. When this is the case, the practitioner should diligently and with persevering effort devote himself to the duty of resuscitating it. Sometimes a gentle compression of the chest or sprinkling water in the face will, by the act of muscular irritation in one instance, or the excitation of the peripheral nerves in the other, cause the contraction of the thoracic muscles, inflating of the chest and a complete respiration. When these are unsuccessful, artificial respiration may be practiced. If there is not a ready response to this the cord should be cut, allowing the proximal end to remain unligated until there has been some bleeding, if indeed it will bleed. Sometimes this will help to start up the circulation of the blood. The face of the child may be again sprinkled with cold water, and if there is yet no response to your efforts, the child should have the nape of the neck and shoulders placed alternately in a basin of hot and cold water. You will find a response to this treatment in many cases. There is another method to which I am very much attached, because in my hands it has been fruitful of many good results. It is to grasp both ankles of the child securely in one hand and place the palm of the other under the shoulders, the fingers extended so as to support the head from the occiput. Now you lower the head to a point in front of or between your feet and elevate the feet in front of you. In placing the child in this position you cause the hands and arms to fall down over the head and in this way expand the chest by pulling on the pectoral muscles. Now, with a long sweep you elevate the hand containing the head, bringing it up quickly, at the same time lowering the other hand. Repeat this movement several times, and if you have never tried it before you will be delighted to see how beautifully it causes the chest to expand, the lungs to fill with air, and respiration to begin. Another method of reviving the asphyxiated is by direct insufflation which is practiced by holding the nose of the child and blowing directly into the mouth. But you will find cases in which none of these will succeed; then you can try them all again, being sure that you do not give up the effort too soon, for thereby valuable lives have been lost. If you ask how long these efforts must be continued, I must say that I cannot answer definitely, but will quote an illustrative case recorded by Dr. A. F. Penrose of Philadelphia. He says: "Thirty-five years ago the wife of a young physician was confined with her first child, under the care of a celebrated professor of obstetrics. The labor was complicated and tedious; the patient during labor and after delivery was in great peril demanding the entire attention of her medical attendant. The child, when born, was apparently dead. The old professor said to the young doctor father (the mother was unconscious and therefore did not hear), "Doctor, cut the cord and take the child away; it is dead, and your wife's condition demands my whole care." The father separated the child, and carried it into the next room and placed it on the bed. He then went back and asked the professor if he was sure the child was dead. Receiving again a positive opinion that the child was dead, and that all attempts to revive it would be useless, the father returned to his dead baby, and having nothing to do, in a wild, hysterical, utterly hopeless sort of way began artificial respiration after the manner I have just described. Half an hour passed with no results; the agonized father continued his efforts. An hour passed, but the infant seemed as hopelessly dead as it was before the artificial respiration was attempted. The man's emotional paroxysms began to subside, and he began to realize that he was literally wasting his breath. Still he did not desist. Suddenly he was startled by a slight, apparently spontaneous movement on the part of the child; with renewed energies he continued his labors and in a short time normal respiration took place, and to his supreme felicity and the astonishment of the medical attendant the child was saved. The great professor is dead; the doctor father is also dead; but the child, called back to life by the hysterical blowings of an agonized father, hopelessly practiced for the very long period of an hour and a half, the child, now a grave, matured man, still lives, the comfort and solace of a mother who that day so nearly died in giving him birth."

Electricity and galvanism have also been suggested, and might be useful in arousing the nerves of a child that was apparently born dead.

The next duty is the bath. In my humble opinion, there is no event in the whole course of human life upon which more depends than the careful and proper administration of the first bath of the new-born babe. I do not believe that it is ever proper for the medical attendant to leave the management of this important matter to any nurse, however competent she may be, without at least supervising it. This should not be considered menial service, and I believe the medical man would reflect credit upon himself and bestow blessings upon humanity if he oftener took charge of this matter himself. In the first place, there should not be a cold room with strong draughts through it, as has often been allowed during the hard pains of labor. The room should have a temperature of 98 or 100 degrees, because if you do not the surface of the tender body will be shocked, the seeds of colds and many other troubles will be sown, and the mother herself is apt to have rigors from too rapid cooling off by the evaporation of the perspiration caused by efforts at expulsive pains. Besides the usual baby basket containing the clothes, you have at hand a soft old rag which you are quite sure has been boiled since it was used for any other purpose; a cake of pure castile soap; an ounce or two of pure hog's lard or vaseline, and a clean, soft, old towel. A basin of warm water, containing bichloride of mercury, of the strength of 1 to 5,000, now completes the outfit. Before beginning the process of washing the baby, the nurse should be very certain that her hands are clean and that she is not contaminated from contact with any communicable disease. Now, with the baby on her lap, in front of the fire, she first anoints the whole surface of the body with the lard or vaseline in order to cause the vernix caseosa to be dissolved and removed by the bath. She then takes her soft rag, and first of all carefully and gently washes the baby's face, taking great pains to cleanse the forehead and around the eyelids. The rag is next folded on itself in such a way as to get a clean portion—a part which has none of the secretion on it—and with this, the margins of the lids being separated, you remove every particle of secretion, and then allow a few drops of water to flush the eye in order to be sure that it is clean and that no germ of venereal disease or any irritant may be left on the conjunctiva to set up trouble. Many cases of purulent ophthalmia have their origin in the conduct of the first bath.

After the eyes have been carefully and properly bathed, the inside of the mouth should be washed; then the whole surface of the body is cleansed with soap and water. Sometimes the vernix caseosa adheres very closely, and it is not necessary to remove every particle of it at the first bath if you must scrub the child forcibly to do it. Better leave the *skin* on it though you leave the adherent secretion with it. A little more lard applied after the bath and before dressing the baby causes it to come away easily at the next bathing. After this you should have another basin of pure warm water, into which the child should be placed and rinsed off, then placed again on the lap of the nurse on a clean, dry apron, and dried off with a soft towel provided for the purpose.

The end of the umbilical stump should now be carefully examined to see that there is no hemorrhage before dressing. Dressing the umbilicus is always to be done with care. If there is any suspicion of gonorrheal matter which may infect the end of the cord, it should be touched with a tablet of bichloride of mercury before being dressed. For dressing the cord a piece of plain sterilized or bichloride gauze, 1 to 1,000, about 4x4 inches in size and two or three thicknesses, having a hole cut in the center through which the cord may be pulled, is as good as anything. When the end of the cord is through the opening of this gauze, which should have been greased with carbolized vaseline before being applied, the corners shall each be folded toward the center, when the dressing is complete and ready for a bandage, to retain it in position. There are a number of different forms of bandages, and I shall not have time in the scope of this paper to go into details on that subject. Suffice it to say, that is best which will best retain the dressing and can be best retained in position, without slipping and getting in an awkward shape and thus become a source of irritation to the child. In all cases it shall be fastened on with small size safety pins. Never use pins with uncovered points, because in handling the child there is great risk of pricking it and thus cause unnecessary suffering.

The child will not be again placed in the bath-tub until after the cord has sloughed off; and this bandage will be unfastened daily in order to anoint the under surface of the dressing with five per cent. carbolized vaseline, which will prevent it from becoming hard and stiff. After the cord is off, the stump shall be sprinkled with a little aristol, subnitrate of bismuth, or some other suitable dusting powder (our grandmothers used charred linen, and as that is dry and antiseptic, we have not made much improvement on it) and the bandage reapplied. This should be worn constantly for three months in all cases, and if the child is cross and fretful, a longer time is necessary in order to protect the umbilicus and prevent abdominal hernia. In dressing the baby only so much clothing as is necessary for its protection should be applied. I wish to enter a protest against the putting of *coarse flannels* next to the skin of new-born babes in midsummer in our climate while the mercury is ranging among the nineties. It produces an entirely unnecessary irritation of the surface, and makes a crying, fretful baby. It is much more sensible to have wraps on the outside of the clothing, where they can be removed at midday, and reapplied morning and evening when found necessary.

After the child has been properly dressed, and the mother has had an hour in which to rest, the child may be applied to the breast. The colostrum which the breast contains is an oleaginous fluid and is a laxative to the baby, and is, in most cases, all that is required. The child shall now be placed in bed, by the mother's side where it can be kept warm, and the child and mother left unmolested, in a perfectly quiet room with very little light, in order to invite the most quiet repose, which is now so necessary.

Visitors shall not be admitted, but the quietude of the apartment shall be preserved until the end of the lying-in period. I wish here to enter my protest against too much attention to the baby, and especially that kind which is bestowed by the average midwife. These meddlesome creatures (I say it with all due respect) are apt to be very busy at this time. One of the first things usually done is to fill the baby's mouth with a sugar teat, then a piece of salty, fat bacon, and when the child has filled its buccal cavity and stomach with this saccharine matter and salty grease it has the *colic*. Now the frolic begins and the helpless little innocent has to submit to being drenched with all manner of teas, decoctions, colic drops, asafetida, and many other things, some of which would tax the digestion of an ostrich. Having finished this task, the *caput succedaneum*, which is so innocent, and does not need her aid, comes in for its share of *shaping up*. This concluded, the little cherub is expected to go off quietly to sleep, and many do so,—into that last long sleep from which they will be awakened at the resurrection morning. Yes, I tell you, it would require cranial bones of steel, stomachs of rawhide, and constitutions of whalebone to resist the treatment which some babies receive. If this society can make operative some restrictions on midwives which will cause them to leave the baby alone after delivery, I believe their greatest evil will be overcome, that mortality tables of new births will be lowered, and that the generation yet unborn will rise up and call you blessed. The child will only require to be applied to the breast two or three times a day for the first day or so, and as a rule will not require any other food until nature's supply is furnished on the third day. The exceptions are few, and cannot be considered further here. The above general suggestions are intended to apply to children born in a normal way at full term. It is beyond the province of this paper to consider injuries that may be done to the head and nervous system in cases of dystocia where the labor has been very long or instruments have been used.

Before leaving the infant at this time, however, we should be perfectly satisfied, by a personal inspection in all cases, that there is no physical deformity or any inperfections without giving to each such attention as it may require. Sometimes there are supernumerary digits which should be removed, or there may be deficiencies of some kind about the genital organs or the alimentary canal. There are some forms of club-foot that are easily remediable by stretching the short muscle every other day for a week or two if done at this very tender age.

### PREMATURE CHILDREN.

Infants that are born prematurely possess small power of resisting external agencies. "The artificial maintenance of the bodily heat is essential to the preservation of life in the early period of extra-uterine existence." The first bath should be given very carefully in water about 100° F., and the child should be warmly wrapped and kept near the fire. But this is a crude and imperfect way of preserving the temperature, the objections to which have been met by the invention of the incubator. Two types of these are represented by the apparatus of Credé and Tarnier. Credé's invention has been used in Maternité at Leipsic for more than twenty years. In this institution the total mortality was eighteen per cent.

His apparatus is a simple tub, shaped very much like an ordinary bath-tub, made of copper with double walls and flooring. The space between these walls is filled with warm water. There is a stop-cock in the bottom, at one end, from which the water is drawn off, and it is refilled at the top once in four hours. The child is placed in this tub, wrapped in fine cotton or soft flannel. The coverings should reach the brim of the vessel, the face of the child alone remaining exposed. The child should only be removed to be bathed and dressed. Tarnier's appliance consists of a double walled wooden box with the space between the walls filled with saw-dust to prevent loss of heat. This is divided by a central partition into an upper and lower compartment. The lower contains a vessel filled with water, and having a space all around it for the free circulation of the air. This water vessel is also connected to a thermo-siphon by an upper and lower tube through which the water circulates when heated by a lamp. There is also another tube at the bottom through which water may be drawn off. The upper compartment is that which contains the baby in its cradle around which the air must circulate freely. This has a cover of two plates of glass and a small opening at each end of the top by which a circulation with the outside is kept up. Now, when the lamp under the siphon is lighted the heated water flows back through the upper tube and establishes a circulation. By the use of the lamp the temperature can be raised to any desired point. In cold weather it has been found necessary to light the lamp three times a day and keep it lighted for about two hours. A registering thermometer placed alongside the child completes the outfit.

A mean temperature of eighty-six is sufficient, but as high as ninety-six is borne without harm. The children should be clothed in the usual manner. "In a two years' trial of this incubator at the Paris Maternité, Auvard reports as follows: Of 93 healthy premature children, 31 died; of 50 premature children with complicated diseases, 15 died." I believe the incubator is fully worthy of further trial and study in rearing premature children.

